# Socioeconomic and household water management determinants of malaria and other vector-borne disease prevention in Urban Gujarat, India

**DOI:** 10.1186/s12936-026-05830-2

**Published:** 2026-02-17

**Authors:** Deepshikha Batheja, Divija Samria, Michael C. Wimberly, Mercedes Pascual, Rajendra Kumar Baharia, Ajeet Kumar Mohanty, Vikas Desai, Keshav Vaishnav, Raj Sharma, Vijay Kohli, Sachin Sharma, Anup Anvikar, Courtney Murdock, Arindam Nandi

**Affiliations:** 1 Indian School of Business, Hyderabad, India; 2 (Formerly) One Health Trust, Bengaluru, Karnataka India; 3 Department of Geography and Environmental Sustainability, University of Oklahoma, Norman, OK USA; 4 Department of Biology and Department of Environmental Studies, New York University, New York, USA; 5 Indian Council of Medical Research-National Institute of Malaria Research, Panaji, Goa India; 6 Indian Council of Medical Research-National Institute of Malaria Research, New Delhi, India; 7 Urban Health and Climate Resilience Centre of Excellence, Surat, Gujarat India; 8 Vector Borne Diseases Control Department, Surat Municipal Corporation, Surat, Gujarat India; 9 (Formerly) Department of Entomology, Ahmedabad, Gujarat India; 10 Department of Entomology, Cornell University, Ithaca, NY USA; 11 Cornell Institute of Host-Microbe Interactions and Disease, Cornell University, Ithaca, NY USA; 12 Center for the Ecology of Infectious Diseases, University of Georgia, Athens, GA USA; 13 One Health Trust, Washington, DC USA; 14 Population Council, 1 Dag Hammarskjold Plaza, New York, NY 10017 USA

## Abstract

**Background:**

Malaria remains a major public health concern in low- and middle-income countries (LMICs), particularly in urban settings experiencing rapid vector adaptation. India, contributing significantly to South-East Asia’s malaria burden, faces persistent urban transmission. While previous studies have explored local determinants of vector-borne diseases, large-scale analyses examining the interplay of socioeconomic factors, water availability, storage practices, and disease prevention behaviors remain limited.

**Methods:**

We conducted a socioeconomic and health survey in the cities of Ahmedabad and Surat, India, between September and November 2023. Data were collected from 4,074 households, comprising 15,484 individuals, and we examined associations between socioeconomic indicators, water availability, storage practices, and mosquito-borne disease incidence and prevention behaviors. Logistic regressions were used to identify significant predictors.

**Results:**

We find that self-reported disease prevalence was low, with 77 cases of malaria, dengue, and chikungunya, equivalent to 18.90 cases per 1,000 households. Socioeconomic factors such as wealth, caste, and family size were significantly associated with disease reporting and prevention practices. Households in the richest wealth quintile were more likely to adopt prevention measures, but less likely to perceive mosquito-related risks. Water management practices, particularly storage in clean large containers, were associated with increased disease symptoms and prevention measures, highlighting the complexity of vector control. Households with impermeable storage containers reported reduced use of active prevention measures.

**Conclusion:**

Socioeconomic disparities and water management practices significantly influence malaria incidence and prevention behaviors. Targeted interventions prioritizing disadvantaged households, improved water storage practices, and enhanced investments in preventive care are essential to reduce vector-borne disease vulnerability and accelerate India’s malaria elimination goals.

**Supplementary Information:**

The online version contains supplementary material available at 10.1186/s12936-026-05830-2.

## Introduction

Malaria remains one of the world’s most significant vector-borne diseases. In 2023, an estimated 263 million malaria cases and 600,000 malaria deaths occurred globally, primarily among children under five in sub-Saharan Africa and rural populations [1]. Caused by *Plasmodium* parasites transmitted via female *Anopheles* mosquito, malaria can result in symptoms including fever, chills, headache, muscle aches, vomiting, diarrhea, and, in severe cases, anemia, jaundice, organ failure, cerebral malaria, or death [2, 3]. India is the largest contributor to the burden of malaria in the World Health Organization (WHO) South-East Asia region, accounting for 66% of the estimated cases and, with Indonesia, 94% of regional deaths in 2022 [4]. Despite significant progress, malaria control has recently stagnated or reversed due to rising insecticide and drug resistance [5–7], funding gaps [8], conflict and humanitarian crises, climate-related disruptions, health-system weaknesses, and inequities that limit access to prevention and treatment [1].

A major emerging challenge is the spread of *Anopheles stephensi* [9, 10], an urban-adapted malaria vector native to South Asia that has recently invaded Africa. *An. stephensi* transmits both *Plasmodium falciparum* and *Plasmodium vivax* parasites and has historically driven large urban malaria outbreaks in India, Iran, Pakistan, and the Arabian Peninsula [11–14]. Its ability to breed in artificial containers like drums, buckets, and construction site pits [15, 16], and its persistence throughout the year, even in dry seasons, make rainfall a poor predictor of transmission [17, 18]. These traits allow the vector to thrive in densely populated urban environments.

With half the world’s population currently living in cities and projections rising 70% by 2050, especially in low- and middle-income countries (LMICs), the WHO has warned of rising threats from urban-adapted mosquito vectors such as *An. stephensi* [4]. Following its detection in Djibouti, more than 2000-fold [19, 20]. Cities in Ethiopia (e.g., Kebri Dehar, Dire Dawa, and Awash) have since detected both *Plasmodium falciparum* and *Plasmodium vivax* in local *An. stephensi* populations [21, 22]. Conditions typical of rapidly growing cities (unplanned migration, high population density, poor housing and sanitation, and stagnant water sites) further amplify malaria transmission [23, 24]. In India, about 10–12% of the total malaria cases arise from urban areas, with the case fatality rate peaking at 21.5 cases per 1000 cases in 2017 [25, 26].

Understanding how socioeconomic factors, water storage practices, and mosquito prevention behaviors shape malaria risk in urban areas of LMICs could provide useful insights for efforts to contain *An. stephensi* in endemic regions. Recent research on malaria in Indian cities has focused on local differences in anthropogenic factors [27–36]. These studies have identified environmental conditions, such as microclimate, land use, and water storage, that foster breeding conditions, and socio-economic factors that limit resources for prevention, treatment, and control measures and may influence riskier behaviors that exacerbate exposure. Research on dengue from Colombia, Guyana, and Nepal also highlights how traditional water storage practices, education levels, and age shape prevention behaviors and disease knowledge patterns [37–39]. These studies have examined community water storage definitions and knowledge, along with attitudes and practices among vulnerable populations, to enhance vector control strategies.

However, the literature has been limited to small sample sizes or narrow geographic focus, often examining such factors in isolation rather than in combination [27–36]. To contribute to this evidence base, we conducted a large household survey across two Indian cities (Surat and Ahmedabad, Gujarat) that continue to experience a seasonal malaria burden despite persistent control efforts and considerable declines since 2019 [27, 28, 40]. To the best of our knowledge, this is the first study to jointly examine the associations between socioeconomic characteristics, water availability and management, perceived mosquito-related risk, prevention behaviors, and self-reported malaria incidence in urban settings.

## Methods

### Study sites

This multicenter cross-sectional study was conducted in the cities of Ahmedabad and Surat in the state of Gujarat between September and November 2023. These cities accounted for 50% of the malaria cases in the state (27% Ahmedabad; 23% Surat) as of January 2023 [41]. The cities are located on the banks of Sabarmati and Tapti rivers, respectively, with annual temperatures ranging from 12 °C to 44 °C, and most rainfall occurring during the monsoon season between June and October [27, 28]. The two cities represent contrasting climates, with Ahmedabad being an inland region with a semi-arid climate and Surat a coastal city with a more humid environment [27]. A 2023 report by the National Institution for Transforming India (NITI) Aayog showed that these cities also housed a total of nearly 11% of multidimensionally poor population [42].

The study was conducted in densely populated urban neighborhoods of Ahmedabad and Surat that are two major Indian cities with estimated populations of 7.6 million and 6.9 million, respectively, in 2025 [43]. The study areas are characterized by compact housing, high population density, municipal piped-water supply, and variable access to sanitation facilities. These areas also fall under active urban vector-control programs and experience seasonal peaks of dengue and malaria transmission, making them relevant settings for examining household-level risk factors.

Gujarat has a strong surveillance program for malaria under the National Vector Borne Disease Control Programme [27]. Specifically, Ahmedabad and Surat have undergone comprehensive and sustained malaria and vector surveillance for more than 20 years [27]. In 2022, Gujarat was reclassified from Category II (between 1 and 2 malaria cases per 1,000 population) to Category I (less than 1 malaria case per 1,000 population) [41]. Despite these efforts, Gujarat has continued to experience intermittent malaria outbreaks in certain villages and urban areas, especially between the months of September and November [27, 28, 40].

### Study design and participants

The household-level survey included 15,484 individuals from 4,074 households, with nearly equal distribution across the two cities to ensure comparable ward coverage and field logistics and to prevent dominance of pooled estimates by a single city. Sample size calculations were informed by state-level malaria surveillance data. Using the three-year annual average for Gujarat reported by the National Vector Borne Disease Control Programme in 2021 (7,732 positive cases in a population of approximately 60 million) [44], we derived an implied prevalence of 0.0129%. Applying a single-proportion formula with 95% confidence (*Z* = 1.96) and a precision of ± 0.05 percentage points yielded a required sample of approximately 1,980 households. To account for cluster sampling and potential intra-cluster correlation, a conservative design effect of 2 was applied, resulting in a target sample of approximately 3,960 households. The final achieved sample of 4,074 households exceeded this target and was adequate to support analyses of associations between socioeconomic factors, household water practices, and prevention behaviors.

The Computer-Assisted Personal Interview (CAPI)-based survey collected data on socioeconomic indicators, household demographics, and health and hygiene practices (see Appendix A). Interviews were conducted with the household head or, if unavailable, an adult household representative knowledgeable about household water use, health, and hygiene practices. The inclusion criteria were the presence of any adult household member (above 18 years of age during surveyor visit) willing to participate, and all interviews were conducted in the local language to ensure clear communication. The data were collected by trained enumerators from a leading survey firm in the country, with site visits and quality checks by the authors and automated logic checks in the CAPI system.

The household questionnaire consisted of five sections (see Supplementary Appendix A): household roster; housing and socioeconomic characteristics; water availability and storage; disease history and recent symptoms; mosquito-prevention practices, treatment-seeking, and expenditures. Each section contained 8–12 primarily close-ended questions with some multiple-response items and built-in skip patterns. Most household characteristics, disease history, symptoms, water availability, and prevention practices were collected through self-reported responses in the questionnaire, whereas water container coverage, container cleanliness, housing structure materials, and the presence of doors/windows/screens were directly observed and recorded by the enumerators. Content validity was ensured through expert review and pilot testing in at least ten households in each city. Any ambiguities identified during pretesting were used for revising the enumeration tools before the main survey.

We used systematic sampling with a target of 2,000 households per city. In Ahmedabad, households were selected from 28 wards (four randomly chosen per zone across seven zones). In Surat, households were spread across 34 wards and nine zones. Sampling began at 92 fixed entomological surveillance sites maintained by local authorities, then continued by selecting every third household to the left until quotas were met. On average, 73 households were surveyed per ward in Ahmedabad and 60 per ward in Surat.

### Outcome variables and independent effects measured

We structure our analysis in three research questions. First, we examined the socioeconomic factors associated with mosquito-borne disease incidence and prevention behaviors, expecting that households with lower socioeconomic status, lower education levels, and larger family sizes would be more vulnerable due to limited access to preventive measures and lower awareness [29, 45–47]. Second, we explored how household water availability and storage practices influence disease risk and prevention behaviors. We hypothesized that water scarcity and improper storage practices would contribute to increased disease risk, while proper storage and purification would reduce vulnerability [15, 37, 48]. Third, we investigated whether perceived mosquito risk influences the adoption of prevention behaviors. We predicted that households with higher perceived mosquito risk would be more likely to use multiple prevention measures, though perceptions might be shaped by actual exposure, socioeconomic status, and prior disease incidence [38, 49].

Informed by prior literature [38, 48, 49], our main outcomes or dependent variables to measure these associations were at least one vector-borne disease case reported during the 90 days preceding the survey (included malaria, dengue, and chikungunya), more than one disease symptom reported, use of active prevention measures, use of more than three prevention measures, use of more than one outdoor prevention measure, and use of more than one indoor prevention measure. From the survey, the self-reported incidence of malaria, dengue and chikungunya, symptoms of mosquito-borne diseases (vomiting, fever, cough, stomachache, or other stomach problems), use of mosquito bite prevention measures, and perceived problem of mosquitos within the household and in the community were converted into binary variables (yes/no). Another binary outcome variable was high risk perception of mosquito problems, defined as below- or above-average perceived risk of mosquito bites.

Active measures were those that require active use of coils, repellents, and nets [38]. Indoor measures were actions taken within the household to reduce mosquito entry or prevent bites. These included structural barriers such as covered doors and windows, the use of bed nets, and personal protection methods, namely, repellents, coils, and other household-based mosquito deterrents [49]. Likewise, outdoor measures refer to community- and neighborhood-level activities aimed at reducing mosquito breeding sites. These included public or private spraying, personal spraying around the premises, application of larvicides in stagnant water, and other community-directed vector control measures [49]. For risk perception, a standardized index was created by adding up response values (ranging from one to four denoting “no mosquitoes” to “a lot of mosquitoes”) to two survey questions on the households’ perception of mosquito problem within and outside the house. The households were then divided between above- and below-mean index values.

Table 1 describes the independent variables of our regression analysis—both the bivariate models presented in Table B.1 and the multivariate models in Tables 5 and 6. Data collected on gender and education of household head, migrant status, religion, and water availability were transformed into dichotomous independent variables. In India, caste signifies a system of social stratification with commonly used categories for population monitoring include Scheduled Castes (SCs), Scheduled Tribes (STs), and Other Backward Classes (OBCs) [50]. Historically marginalized, SCs and STs experience substantial disparities in access to health care and health outcomes [51, 52]. These three categories were retained in the analysis. Other computed independent variables included household wealth index, and cleanliness and permeability of water storage containers. Descriptive statistics informed the creation of derived independent variables by accounting for distribution of the data. For skewed distributions, median was used to parse the data instead of the mean.

**Table 1:** Description of independent variables

Variable	Type	Description
Household characteristics		
Age of household head	Numeric	–
Education of household head	Binary	0 = Below High School (Class 6 to 10) 1 = Above High School
Family size	Numeric	Number of family members residing in the house
Proportion of females	Percentage	Including both adults and children
Proportion of children	Percentage	Household members below 18 years of age
Migrant status	Binary	0 = From the same city 1 = Not from the same city
Caste	Categorical	0 = General 1 = Scheduled Caste 2 = Scheduled Tribes 3 = Other Backward Classes
Religion	Binary	0 = Majority (Hindu) 1 = Others (Islam, Buddhism, Christianity, Sikh)
Wealth index	Categorical	0 = Bottom 20 percent (0–20%) 1 = Bottom 40 percent (20–40%) 2 = Bottom 60 percent (40–60%) 3 = Top 40 percent (60–80%) 4 = Top 20 percent (80–100%)
Protection behavior		
Expenditure on cleanliness	Numerical	Log (1 + money spent on household cleanliness and personal hygiene)*
Water availability		
Less water availability	Binary	0 = Water available more than once a day 1 = Water available only once a day
Relative frequency of fetching water	Binary	0 = Low/below-average frequency 1 = High/above-average frequency
Water storage practices		
Purification and storage	Binary	0 = Household either does not purify, does not store water or both 1 = Household purifies and stores water
Type of water storage container	Categorical	1 = None 0 = Only small containers** 2 = Both small and large, or only large containers
At least one large container is clean	Binary	0 = Otherwise 1 = At least 1 large container is cleaned > 2 times a year
At least one small container is clean	Binary	0 = Otherwise 1 = At least 1 large container is cleaned > 4 times a year
At least 1 large container is impermeable	Binary	0 = Container is permeable 1 = Container is covered fully using a lid and mosquitoes cannot enter
At least 1 small container is impermeable	Binary	0 = Container is permeable 1 = Container is covered fully using a lid and mosquitoes cannot enter

*This log transformation was applied to reduce skewness of the variable and account for values which are 0

**The categories are not in ascending order to maintain ‘only small containers’ as reference group in the regression analysis

The wealth index divided the households into five wealth quintiles, quintile 1 being the poorest 20% and quintile 5 being the wealthiest 20% of households, based on a principal component analysis (PCA) method as described in the Indian Demographic and Health Surveys (DHS) for 2019–2021 [53]. We included three categories of wealth indicators as verified by the enumerators in the PCA index to create the wealth quintiles. The first category captured household assets, including the presence of a detailed list of 28 items such as television, radio, bike etc. The second category reflected household structure and quality, covering house ownership (owned or rented), material used in the construction of house roof and walls (concrete versus others), classification of house as “kutcha” (temporary or semi-permanent structure) versus “pucca” (permanent structure built with strong, durable, and stable material), single-storied versus multiple-storied dwelling, number of rooms and floors in the house, and the presence of a separate kitchen. The third category related to water and sanitation facilities, including the primary water source (own house tap versus others), type of toilet (flush versus others), and whether the toilet was shared with other households.

Local government authorities advise households to clean large water tanks (e.g., on the rooftop) once every six months and small containers as frequently as possible, with the questionnaire capturing cleaning up to four times a year [54]. These thresholds were used to determine whether households cleaned their containers regularly. A container was permeable if it was partially covered, uncovered, or if mosquitoes could enter regardless of coverage. All other containers were considered impermeable. These indicators of cleanliness and coverage were included as covariates of the regression model.

### Statistical analyses

We conducted separate logistic regressions for each of these outcome indicators described above on each independent variable of socio-economic household characteristics, water availability, and water management practices among others. To determine statistically significant associations between dependent and independent variables, bivariate regression analysis was performed. Consistent with prior research on socio-economic and household determinants of vector-borne diseases [55, 56], variables that showed a statistically significant association at the 5% level were then included in the multivariate regression models. This approach ensured that only those independent variables with a meaningful effect on the dependent variable are considered in the multivariate analyses while accounting for potential confounding effects from other variables.

In the multivariate regressions, two models were evaluated: one model with only household background characteristics (e.g., demographics and standard of living) and one with the full set of independent variables discussed below to understand how the associations evolved. Regression estimates were reported with standard errors clustered at the city ward level. Resultant odds ratios that were significant at 95% confidence level (p-value < 0.05) are reported from the multivariate logistic regressions. In our models, the month fixed effects account for time trends and factors unique to each survey month (e.g., seasonal variation in disease patterns), while city fixed effects control for unobservable characteristics within each city that are constant over time (e.g., healthcare infrastructure), which might affect vector-borne disease incidence and control. Statistical analyses were conducted using Stata software (version 16.0).

## Results

### Descriptive statistics

#### Household characteristics

Table 2 summarizes the socio-economic characteristics of the 4,074 surveyed households (15,848 individuals). Households had an average of four members, with 47% female and 53% male individuals, including both children and adults. Household heads were predominantly male (86%) with a mean age of 47 years. Adults and children had average ages of 38 and 10 years, respectively.

**Table 2:** Household characteristics

	Frequency (n)	Sample proportion
Number of households	4,074	
Number of individuals	15,484	
City		
Ahmedabad	2,036	49.98%
Surat	2,038	50.02%
Gender (including children)		
Female	7,257	46.88%
Male	8,222	53.12%
Other	5	0.12%
Gender of household head		
Female	583	14.31%
Male	3,491	85.69%
Average age (in years)		
Adults	38.37	
Children	9.78	
Average age of household head (in years)	46.86	
Education of household head		
No education or below High School (class 6–10)	1,484	36.43%
High School or higher	2,590	63.57%
Primary source of income		
Agricultural wage labor	42	1.03%
Non-agricultural wage labor	375	9.2%
Own cultivation	25	0.61%
Salaried employment	2,239	54.96%
Self-employment	303	7.44%
Trade/business	1,024	25.14%
Pension/rent/dividend	66	1.62%
Migrant status		
From the same city	3,323	81.57%
Not from the same city	751	18.43%
Religion		
Hindu	3,816	93.69%
Islam, Christianity, Buddhism, Sikhism	257	6.31%
Caste*		
General	1,282	33%
Scheduled Castes	718	18.48%
Scheduled Tribes	1368	35.21%
Other Backward Classes	517	13.31%

Data are sample size (n) and sample proportion (%)

^*^189 households or 4.64% of the sample either did not know or did not want to reveal

Regarding education, 36.43% of household heads had no formal education or had studied up to Class 10, while 63.57% had completed high school or higher. The primary source of income varies across households, with the largest proportion engaged in salaried employment (54.96%), followed by trade or business (25.14%). Most households (80%) were native residents of the city, while 18.43% were migrants. Disadvantaged caste groups (SC, SC, and OBC) comprised 67% of the sample (Table 3).

**Table 3:** Self-reported disease burden

Disease/Symptom	Sample households (n = 4,074)	Sample individuals (n = 15,484)
Malaria	54 (1.33%)	62 (0.40%)
Dengue	24 (0.59%)	26 (0.17%)
Chikungunya	2 (0.05%)	2 (0.01%)
Vomiting	86 (2.11%)	116 (0.75%)
Fever/Cough	925 (22.70%)	1,144 (7.39%)
Stomach-ache or other stomach problems	167 (4.10%)	232 (1.50%)

Data are number (n) and proportion (%) of sample households and individuals

#### Water availability and storage

The primary source of drinking water was their own house tap, which supplied water provided by the government, although only 13% reported continuous, all-day water availability. Among those with intermittent water supply, about 75% of sample households received water for two to three hours a day. For drinking water, 51% of households in Ahmedabad and 37% in Surat stored water, but only 67% and 35% of households purified stored water, respectively. The most popular storage method for drinking water was covered vessels, mainly water bottles and utensils.

**Fig. 1:**
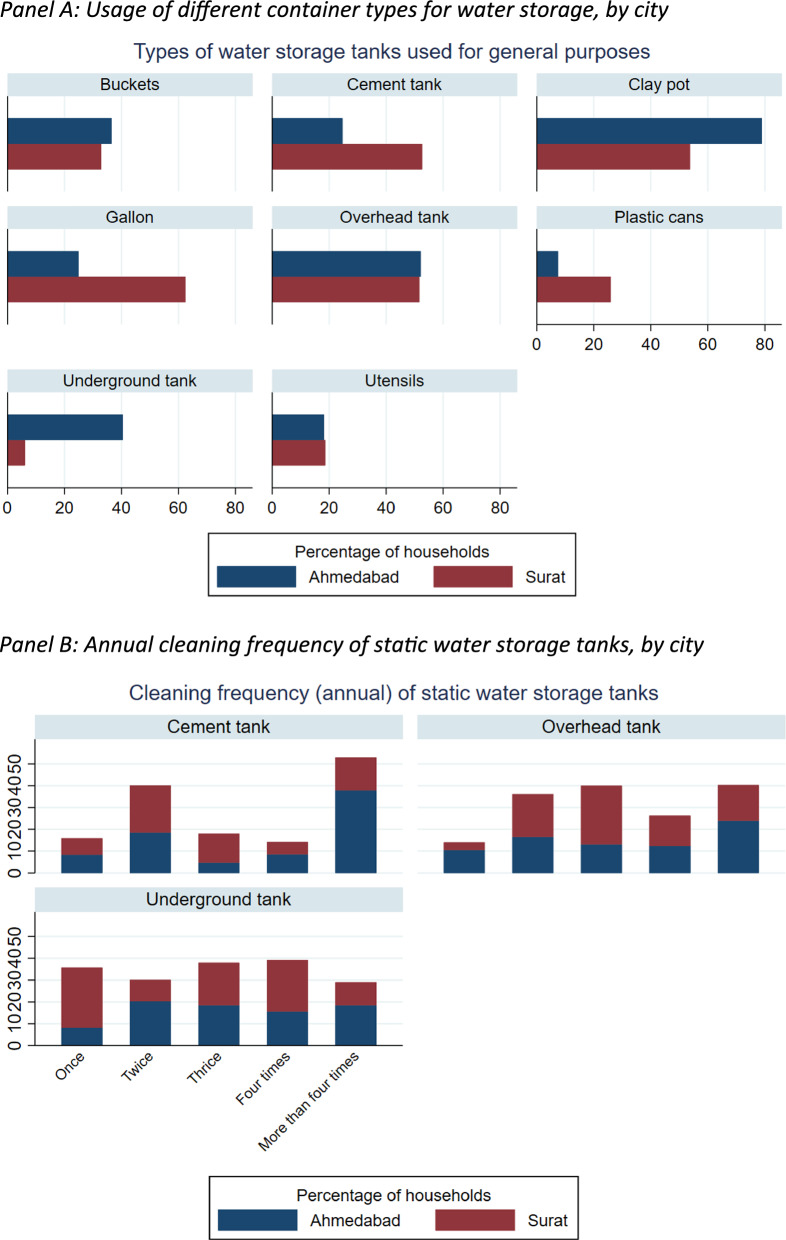
City-wise storage and cleaning behavior of containers used for general purpose water. Panel **A** Usage of different container types for water storage, by city. Panel **B** Annual cleaning frequency of static water storage tanks, by city. Data shows percentage of sample households

For non-drinking purposes, 96.2% of households stored water. Figure 1 summarizes overall patterns in water storage and container maintenance across sample households. The most commonly used containers for general-purpose water storage were clay pots, utensils, overhead tanks, and cement tanks, showing a reliance on a mix of small, movable containers and large static tanks. Cleaning practices varied by container type: cement tanks were cleaned most frequently, while underground tanks were generally cleaned once or twice a year, and overhead tanks showed moderate cleaning frequency. Although variations existed between Ahmedabad and Surat, the key finding is the broad heterogeneity in container use and cleaning frequency in the overall sample, which has direct implications for mosquito-breeding risk regardless of city. Across both cities, more than 75% of households believed that mosquitoes could not enter cement and underground tanks, suggesting a perception that these containers provide greater protection against mosquito entry.

#### Disease prevalence, treatment and prevention

A total of 77 households (1.89%) reported at least one mosquito-borne disease in the 90 days preceding the survey, corresponding to 18.9 cases per 1,000 households and 4.97 cases per 1,000 population. These households reported a total of 80 disease episodes, as some experienced more than one illness. Of these, 54 were malaria (67.5%), 24 were dengue (30.0%), and 2 were chikungunya (2.5%). When accounting for all reported episodes, the overall burden was 19.6 disease reports per 1,000 households and 5.2 reports per 1,000 population. In both cities, the number of households reporting disease cases was relatively low, although there was considerable reporting of fever or cough, followed by stomach problems and vomiting. Table 4 shows that for households that reported cases, nearly all sought treatment. Treatment expenditure for dengue was highest (above INR 4,300 i.e., approximately USD 52) in both cities compared to malaria and chikungunya. Hospitalization rates were also higher for dengue in both cities (85.7% in Ahmedabad; 77.8% in Surat), compared with malaria (42.5% and 38.5%, respectively).

**Table 4:** Treatment for reported diseases

Disease	Ahmedabad	Surat
Treatment taken (%)		
Malaria	40 (97.56%)	13 (100%)
Dengue	14 (93.33%)	9 (100%)
Chikungunya	2 (100%)	–
Hospitalization (%)		
Malaria	17 (42.5%)	5 (38.46%)
Dengue	12 (85.71%)	7 (77.78%)
Chikungunya	2 (100%)	–
Cost incurred (INR)		
Malaria	2,298.75	1,773.08
Dengue	4,914.29	4,388.89
Chikungunya	3,750.00	

Data are number (n) and proportion (%) of sample households that reported a disease case and average cost incurred

*INR* Indian National Rupees

**Fig. 2:**
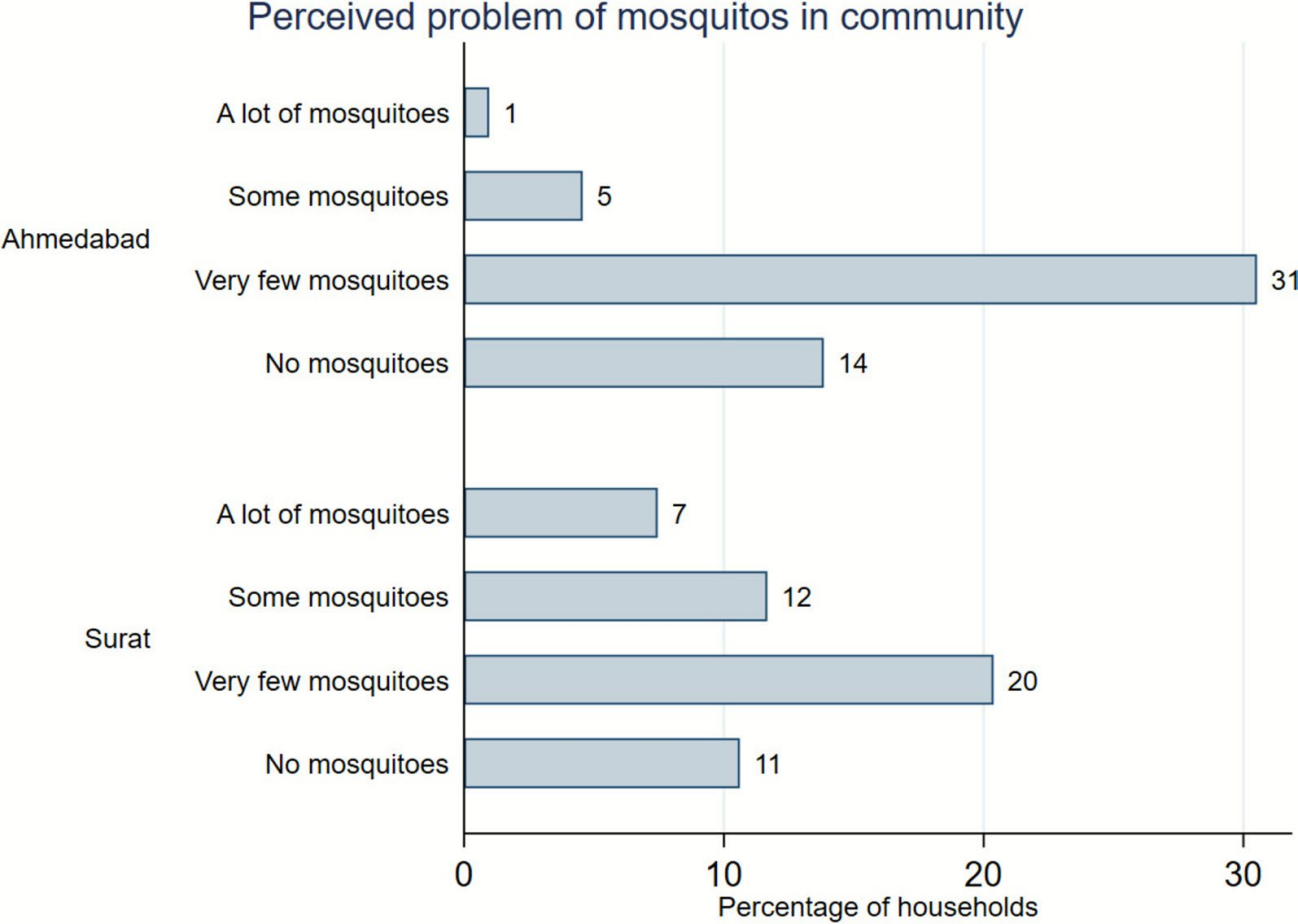
City-wise risk perception of mosquito problem in community. Data shows percentage of sample households

**Fig. 3:**
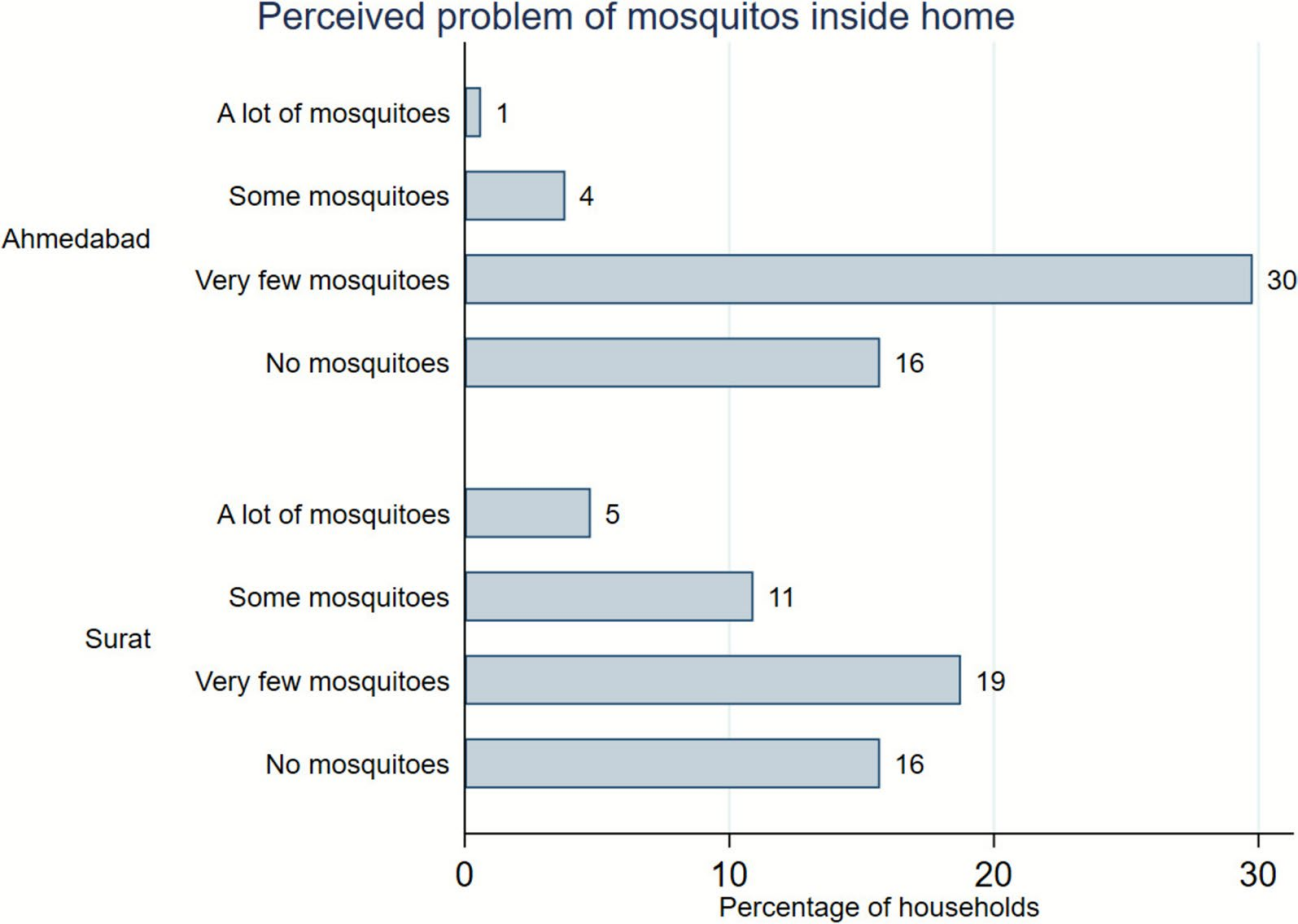
City-wise risk perception of mosquito problem inside the house. Data shows percentage of sample households

Figures 2 and 3 indicate generally low perceived mosquito problems in both cities. About 45% and 31% of households in Ahmedabad and Surat, respectively, reported very few or no mosquitoes in the community. Similarly, 46% and 35% of households in Ahmedabad and Surat, respectively, perceived very few or no mosquitoes within their homes. Environmental spraying by the government was the most commonly reported neighborhood-level mosquito prevention measure. In addition, some households used personally spraying and larvicides, particularly in areas where government spraying was reportedly less common. Within households, covering doors and windows was the most prevalent prevention measure, followed by the use of coils or vaporizers (e.g. All Out brand).

### Regression analysis

The results of the bivariate regression model are presented in Appendix B (Tables B.1 and B.2) and highlight significant (p-value < 0.05) predictors of disease reporting, prevention measures, and mosquito risk perception. Older household heads, larger families, and higher wealth were related to higher disease reporting and greater use of prevention behaviors. Higher education was tied to lower risk perception. SC/ST households reported more symptoms but used fewer prevention measures, while migrants reported fewer symptoms but more disease cases. Restricted water availability was associated with increased use of active prevention measures, while water purification practices and having impermeable storage were linked to reduced prevention behaviors and lower risk perception. Clean water containers were correlated with higher disease reporting and greater prevention use.

#### Multivariate Model I

Table 5 presents the results of the multivariate logistic regressions with only socio-economic characteristics of households. Higher family size was associated with higher likelihood of self-reported disease incidence and symptom prevalence. The likelihood of more than one symptom prevalence increased with the increase in proportion of children in the household (aOR: 1.019, 95% CI: 1.006–1.032, *p* < 0.01). Household wealth was also significantly associated with both self-reported disease and symptom prevalence. Wealthier households (top 20% wealth quintile) had significantly higher odds of reporting disease incidence (aOR: 3.37; 95% CI: 1.131–1.509, *p* < 0.01) compared to the poorest 20%. Meanwhile, households in the bottom 40% (aOR: 0.497; 95% CI: 0.261–0.946, p < 0.05) and bottom 60% (aOR: 0.161; 95% CI: 0.061–0.422, *p* < 0.01) had significantly lower odds of reporting symptoms.

**Table 5:** Multivariate logistic regression results (Model I), adjusted odds ratios (aOR)

	(1)	(2)	(3)	(4)	(5)	(6)	(7)
	At least 1 disease case reported	More than 1 symptom reported	Active prevention measure used	More than 3 prevention measures used	More than 1 indoor measure used	More than 1 outdoor measure used	High risk perception of mosquitoes
Model (1)							
Demographics							
Age of household head (in years)		0.995		0.991**		0.991	
		(0.010)		(0.004)		(0.007)	
Education of household head							
Below High School		Ref				Ref	Ref
High School and above		2.117*				1.459**	1.134
		(0.945)				(0.263)	(0.175)
Family size	1.306***	1.257***				0.860*	
	(0.096)	(0.107)				(0.067)	
Proportion of female members				0.993***		0.998	
				(0.002)		(0.002)	
Proportion of children		1.019***				0.991**	1.004
		(0.007)				(0.004)	(0.003)
Wealth index							
Bottom 20 percent (0–20%)	Ref	Ref		Ref	Ref	Ref	Ref
Bottom 40 percent (20–40%)	1.213	0.497**		3.447***	3.551***	3.805***	0.847
	(0.531)	(0.163)		(0.870)	(0.945)	(1.124)	(0.177)
Bottom 60 percent (40–60%)	1.591	0.161***		5.136***	2.996***	5.094***	0.873
	(0.665)	(0.079)		(1.405)	(0.873)	(1.635)	(0.220)
Top 40 percent (60–80%)	0.683	0.538		6.081***	4.007***	6.349***	0.923
	(0.374)	(0.253)		(1.732)	(1.178)	(2.049)	(0.243)
Top 20 percent (80–100%)	3.368***	0.701		3.236***	2.936***	6.189***	0.599
	(1.388)	(0.273)		(1.079)	(1.010)	(2.388)	(0.215)
Migrant							
From the same city		Ref					Ref
Not from the same city		0.401**					2.691***
		(0.181)					(0.830)
Caste							
General		Ref		Ref	Ref	Ref	Ref
Scheduled Castes		6.223***		1.270	0.748	0.612	1.940*
		(3.458)		(0.455)	(0.266)	(0.255)	(0.690)
Scheduled Tribes		2.478*		0.435***	0.470**	0.181***	0.722
		(1.334)		(0.135)	(0.150)	(0.075)	(0.270)
Other Backward Classes		0.911		0.572**	0.856	0.320***	2.100***
		(0.402)		(0.144)	(0.248)	(0.114)	(0.537)
Religion							
Majority (Hindu)			Ref				
Others			1.350				
			(0.390)				
Month Fixed Effects	Yes	Yes	Yes	Yes	Yes	Yes	Yes
City Fixed Effects	Yes	Yes	Yes	Yes	Yes	Yes	Yes
Observations	4074	3885	4073	3885	3885	3|885	3885
Wald Chi^2	38.91	174.9	9.493	100.4	64.27	130.9	68.59
Prob > chi2	0	0	0.0499	0	0	0	0
Adj R2	0.077	0.158	0.026	0.01	0.079	0.184	0.124

Standard errors clustered at the ward level

*Ref* Reference category

^***^ p < 0.01, ** p < 0.05, * p < 0.1

Additionally, SC households had significantly high odds of reporting disease symptoms (aOR: 6.222; 95% CI: 2.094–18.494,* p* < 0.01) and OBC households had a higher risk perception of mosquito problems (aOR: 2.100; 95% CI: 1.272–3.468,* p* < 0.05) compared to the General category households. Among migrant households, there was less likelihood of reporting symptoms (aOR: 0.401; 95% CI: 0.166–0.970,* p* < 0.05), but a higher risk perception of mosquitoes (aOR: 2.691; 95% CI: 1.470–4.924, *p* < 0.01).

Households with older household heads (aOR: 0.991; 95% CI: 0.982–0.999, *p* < 0.05) and more female members (aOR: 0.993; 95% CI: 0.989–0.998,* p* < 0.05) were significantly less likely to use more than three prevention measures. Expectedly, households with educated household heads were more likely to use outdoor mosquito prevention measures (aOR: 1.459; 95% CI: 1.025–2.077, *p* < 0.05), but households with greater proportion of children were less likely to do so, although only by a small margin (aOR: 0.991; 95% CI: 0.983–0.999, *p* < 0.05). Wealth status was also strongly associated with usage and diversity in prevention measures, i.e., wealthier households had significantly higher odds of using more than three prevention measures, more than one indoor and more than outdoor measures. On the other hand, households belonging to backward castes, specifically STs and OBCs, were less likely to use such mosquito prevention measures.

#### Multivariate Model II

Table 6 shows the results of multivariate analyses with water availability and management practices along with the independent variables included in Model I. For outcomes on disease and symptom prevalence, and usage of prevention measures, the significance and direction of associations observed in Model I were unchanged with the addition of other predictors, but the magnitude of association reduced. However, more prominently, significant associations with wealth quintiles with usage of prevention measures did not hold in this model, indicating that water availability and storage practices were strongly correlated with household wealth and potentially confounding our results in Model I.

**Table 6:** Multivariate logistic regression results (Model II), adjusted odds ratios (aOR)

	(1)	(2)	(3)	(4)	(5)	(6)	(7)
	At least 1 disease case reported	More than 1 symptom reported	Active prevention measure used	More than 3 prevention measures used	More than 1 indoor measure used	More than 1 outdoor measure used	High risk perception of mosquitoes
Model (II)							
Demographics							
Age of household head (in years)		0.991		0.994		0.991	
		(0.008)		(0.005)		(0.007)	
Education of household head							
Below High School		Ref				Ref	Ref
High School and above		1.375				0.863	1.132
		(0.391)				(0.155)	(0.161)
Family size	1.245***	1.248***				0.962	
	(0.091)	(0.099)				(0.093)	
Proportion of female members				0.994**		0.998	
				(0.003)		(0.003)	
Proportion of children		1.014**				0.989**	1.005*
		(0.006)				(0.005)	(0.003)
Wealth index							
Bottom 20 percent (0–20%)	Ref	Ref		Ref	Ref	Ref	Ref
Bottom 40 percent (20–40%)	1.262	0.366***		1.095	1.107	2.061*	0.815
	(0.536)	(0.108)		(0.387)	(0.376)	(0.768)	(0.157)
Bottom 60 percent (40–60%)	1.642	0.181***		1.336	0.687	2.973**	0.752
	(0.679)	(0.095)		(0.503)	(0.282)	(1.335)	(0.181)
Top 40 percent (60–80%)	0.702	0.516		1.677	1.079	2.730**	0.721
	(0.371)	(0.264)		(0.662)	(0.458)	(1.236)	(0.175)
Top 20 percent (80–100%)	2.484**	0.511		0.824	0.757	2.188*	0.526**
	(1.013)	(0.233)		(0.344)	(0.335)	(0.973)	(0.163)
Migrant							
From the same city		Ref					Ref
Not from the same city		0.308***					3.113***
		(0.106)					(1.064)
Caste							
General		Ref		Ref	Ref	Ref	Ref
Scheduled Castes		5.476***		0.862	0.497*	0.547	2.219**
		(2.540)		(0.296)	(0.186)	(0.222)	(0.873)
Scheduled Tribes		3.958***		0.360***	0.403***	0.150***	0.799
		(2.095)		(0.117)	(0.142)	(0.063)	(0.309)
Other Backward Classes		1.078		0.462***	0.715	0.204***	2.592***
		(0.493)		(0.131)	(0.232)	(0.092)	(0.698)
Religion							
Majority (Hindu)			Ref				
Others			1.261				
			(0.334)				
Protection behaviors							
Expenditure on cleanliness (in INR)						1.140	
						(0.251)	
Water availability							
Less water availability							
Water available more than once a day		Ref	Ref				
Water available only once a day		0.486	1.459				
		(0.215)	(0.351)				
Relative frequency of fetching water							
Low/below-average frequency		Ref					
High/above-average frequency		2.387***					
		(0.661)					
Storage practices							
Household purifies and stores water		1.607	0.587**				0.396***
		(0.553)	(0.142)				(0.115)
Water storage container type							
None/Do not store		2.106	1.750**				1.537
		(1.235)	(0.393)				(0.416)
Only small		Ref	Ref				Ref
Both small and large or only large		-	5.366***				-
			(2.552)				
Large container is clean							
Otherwise		Ref	Ref	Ref		Ref	
At least 1 large container is clean		2.174**		1.834**		3.214***	
		(0.682)		(0.492)		(1.075)	
Small container is clean							
Otherwise	Ref						Ref
At least 1 small container is clean	2.221***						2.648***
	(0.507)						(0.600)
Large container is impermeable							
Large container is permeable				Ref	Ref	Ref	
At least 1 large container is impermeable				0.462**	0.555**	0.431*	
				(0.146)	(0.166)	(0.199)	
Small container is impermeable							
Small container is permeable						Ref	
At least 1 small container is impermeable						0.743	
						(0.223)	
Month Fixed Effects	Yes	Yes	Yes	Yes	Yes	Yes	Yes
City Fixed Effects	Yes	Yes	Yes	Yes	Yes	Yes	Yes
Observations	3919	3732	4073	2533	2533	2102	3732
Wald Chi^2	56.26	236.6	36.91	46.65	33.53	154.9	135.7
Prob > chi2	0	0	0	0	0	0	0
Adj R2	0.075	0.233	0.058	0.074	0.0712	0.239	0.160

Standard errors clustered at the ward level

*Ref* Reference category

^***^ p < 0.01, ** p < 0.05, * p < 0.1

Among those who store water, cleanliness of small containers (aOR: 2.221; 95% CI: 1.419–3.475, *p* < 0.05) was positively associated with prevalence of mosquito-borne diseases, while cleanliness of large containers (aOR: 2.174; 95% CI: 1.176–4.020, *p* < 0.05) was positively associated with prevalence of disease symptoms. Households that purify and store water had lower odds of using active prevention measures (aOR: 0.587; 95% CI: 0.365–0.944, *p* < 0.05), but households that store water using large containers had higher odds of using these active measures (aOR: 5.366; 95% CI: 2.113–13.627, *p* < 0.01). On the other hand, households that did not store water regardless of purification had 75% higher odds (aOR: 1.750; 95% CI: 1.127–2.717, *p* < 0.05) of using active prevention measures. Households that frequently cleaned their large containers had a higher likelihood of using more than three prevention measures (aOR: 1.834; 95% CI: 1.084–3.104, *p* < 0.05) and outdoor measures (aOR: 3.214; 95% CI: 1.669–6.191, *p* < 0.01). However, households with impermeable large containers (i.e., with adequate coverage such that mosquitoes did not enter) were less likely to use multiple prevention measures, both indoors and outdoors.

Associations of risk perception with migrant status and caste increased in magnitude in Model II. Wealth was significantly associated in Model II; households belonging to the top 20% wealth quintile also had 47.4% lower odds (aOR: 0.526; 95% CI: 0.286–0.967, *p* < 0.05) of having higher perceived risk from mosquitoes. Similar to usage of prevention measures, households that purify and store water were less likely (aOR: 0.396; 95% CI: 0.225–0.699, *p* < 0.01) to have higher perceived risk, and households with cleaned small containers were more likely (aOR: 2.648; 95% CI: 1.698–4.130, *p* < 0.01) to have a higher perceived risk.

## Discussion

This study evaluated the associations of socio-economic indicators and household water management practices with mosquito-borne disease incidence, symptom prevalence, use of prevention measures, and the perception of disease risk. Overall, the results underscore how social disadvantage, household composition, and water-related behaviors jointly shape vulnerability, consistent with prior work identifying malaria as a disease closely linked to poverty and constrained living conditions [47]. Larger family size and households with a higher proportion of children were linked to lower use of preventive measures, likely reflecting financial constraints and competing caregiving demands. Wealthier households showed higher reported disease incidence, possibly indicating better access to diagnostic services and awareness, rather than greater susceptibility. Other common pathways explored in the literature include access to antimalarials, health-seeking behaviors, housing quality and food security [47]. The composition and size of the household can influence age-specific disease transmission, the use of shared spaces, especially in Indian urban landscapes [27, 30, 45, 57].

When indicators of water availability and management practices were included in the model, the associations generally persisted, but with reduced magnitudes, suggesting that water-related conditions partially mediate or confound socioeconomic patterns. This was particularly evident for wealth and prevention-measure use, where associations weakened substantially once water practices were added. Conversely, households in the richest wealth quintile were less likely to perceive mosquito-related risks, suggesting that, beyond water practices, other socioeconomic advantages such as housing construction may contribute to a sense of perceived protection. Similarly, the relationships between risk perception and migrant status, as well as caste, became more pronounced, implying that these groups may be more affected by inadequate water infrastructure.

The significant associations between disadvantaged castes and heightened vulnerability to mosquito-borne diseases and symptoms mirror the disproportionately high disease burden on these communities in national data such as the Longitudinal Ageing Study in India (LASI) and the National Vector Borne Disease Control Programme surveillance data (NVBDCP) [46, 58]. Our estimates suggest these households were less likely to use prevention measures despite a higher risk perception of mosquito problems. Migrant households and marginalized caste groups may have different risk perceptions due to variations in prior malaria exposure, differential access to health information, poorer housing and occupational conditions, and lower engagement or trust in health services. This calls for a closer look at social and structural barriers that percolate health behaviors and systems in Indian cities in ways such as residential segregation and unmet need for healthcare [51, 59]. For instance, a 2023 study in the city of Bengaluru, India, showed how differences in water governance affect mosquito burdens, with more influential households accessing better vector control [60].

Water availability and management practices were also key determinants of disease and symptom prevalence. We found a strong link between water scarcity and higher symptom reports, which is in line with studies linking frequent water fetching and use of containers to mosquito breeding [61]. Households that reported purifying stored water showed lower perceived mosquito risk and households with impermeable storage containers reported reduced use of active prevention measures. The two findings suggest that water purification and container coverage may function as complementary prevention measures adopted alongside routine water storage [62].

Interestingly, having cleaned small and large containers was associated with higher disease cases and symptoms, respectively. This counterintuitive pattern could suggest the complex role of clean, man-made water containers as breeding sites for *An. stephensi* in urban areas, as documented in past entomological work [4, 15]. Alternatively, this could be a case of reverse causality if households cleaned containers more frequently in response to illness. These same households were also more likely to use active prevention strategies, hinting that neighborhood-level mosquito abundance drives both greater disease reporting and more intensive household prevention behavior [49]. These findings underscore the importance of integrating water management, particularly container maintenance and coverage, into household-level and municipal vector control measures [63, 64].

Our study has important policy implications. Historically, the need for distinctive intervention strategies in curbing urban malaria was recognized in India’s Urban Malaria Scheme as early as 1971. The scheme aims at reducing transmission and morbidity through parasite and vector control, especially implemented through civic body byelaws, targeting urban areas with Annual Parasitic Incidence (API) above two cases per 1,000 population [65]. This scheme continues to be implemented in 131 towns as part of the ambitious National Framework for Malaria Elimination, which seeks to eradicate the disease by 2030, with recommendations from the 2022 Malaria Programme Review (MPR) [66]. A key gap identified by the MPR was a lack of ‘economic-epidemiological’ analyses and the need to understand social determinants in urban settings [67]; our study contributes evidence to this gap.

Disease control interventions should prioritize low-income households, larger families, and households with many children—groups shown here to face higher susceptibility. For disadvantaged caste communities, strengthening access to prevention and timely treatment (e.g., Artemisinin-based Combination Therapy or ACT in tribal areas) remains essential [58]. Targeted city-level interventions, such as larvicide, indoor residual spraying, habitat reduction, thermal fogging, community-level water governance, and disease surveillance can help reduce risk among at-risk populations. Our findings emphasize the need for continued investment in these preventive strategies.

## Limitations

This study has certain limitations. First, the study relied on self-reported disease and symptom cases, which introduces the potential for recall bias or inaccurate reporting. Second, the variable capturing ‘more than one disease symptom’ was constructed from general symptoms (diarrhea, vomiting, fever or cough, and stomach problems) that are not specific to vector-borne or waterborne diseases. Although we used the count of more than one symptom rather than individual symptoms to increase relevance, this measure may still lack disease specificity. Third, risk perceptions may vary depending on recent disease exposure, which may not be fully captured in the survey. Fourth, using fixed entomological surveillance sites as starting points may have introduced selection bias. As part of malaria surveillance programs, these households may be more likely to adopt preventive measures, report cases or have different disease patterns, possibly biasing the results, although these households comprised only 2.3% of the sample. Lastly, other confounders such as local health infrastructure quality, environmental variations, or community-level interventions may also influence outcomes.

We overcame some of the study limitations using multiple techniques. First, the survey was conducted between September and November, which coincides with the transmission season for malaria, dengue and chikungunya. This helps reduce underestimation of self-reported data and increases the likelihood of recent exposure to diseases not only at the household level but also across communities. Second, unlike many cross-sectional studies, the wealth index in this analysis incorporated a comprehensive list of wealth indicators, consisting of asset ownership, household characteristics, and access to water and sanitation. The PCA statistical method retained the most important variations, while enumerator verification added credibility to self-reported data.

## Conclusion

This study demonstrates the interplay between socioeconomic factors and water management practices in shaping vulnerability to mosquito-borne diseases, including malaria, in urban settings. Addressing these factors through improved water purification and storage practices, community-level vector control, and targeted support for socioeconomically disadvantaged households may help reduce disease risks. Sustained investments in preventive care and targeted interventions are critical to achieving India’s malaria elimination goals.

## Supplementary Information

Additional file 1.

Additional file 2.

## Data Availability

Data are available from the corresponding author on reasonable request.
