# Supplementary material for: Socioeconomic and household water management determinants of malaria and other vector-borne disease prevention in Urban Gujarat, India
Source: Malar J. 2026 Feb 17;25:141. doi: 10.1186/s12936-026-05830-2 (PMC13015023; doi:10.1186/s12936-026-05830-2)
Supplement: Supplementary file 1 — Additional file 1. [file 12936_2026_5830_MOESM1_ESM.pdf]

Supplementary Appendix A

Appendix A: Interview questionnaire with translations in the local language

Household Questionnaire (ઘર-પરિવાર માટેની)

P1.	Name of the Head of the household<br>ઘર-પરિવારના વડાનું નામ						
P2.	Address (સરનામું)						
P2.1	Household no (ઘર નંબર)						
P2.2	Locality (વિસ્તાર)						
P2.3	Landmark (નજીકની કોઈ મોટી નિશાની)						
P2.4	Contact no. of the Head of the household<br>(ઘરના વડાનો સંપર્ક નંબર)						
Interviewer: COMPLETE BEFORE BEGINNING THE MODULE<br>(ઈન્ટરવ્યુઅર મોડ્યુલ શરૂ કરતા પહેલા ભરો)							
P3.	Interviewer Code (ઈન્ટરવ્યુઅર કોડ)						
P4.	Supervisor Code (સુપરવાઇઝર કોડ)						
P5.	District Name (જિલ્લાનું નામ)	Ahmedabad			1		
		Surat			2		
P6.	PSU Code						
P7.	Household ID Number<br>(ઘર-પરિવારનો આઇડી નંબર)						
P8.	Date of Interview (DD-MM-YY)<br>(ઈન્ટરવ્યુની તારીખ)					2	3
		D	D	M	M	Y	Y
P9.	Interview start time (ઈન્ટવ્યુ શરૂ કર્યાનો સમય)						
		H	H	M	M	AM	PM

Block A: Household Roster for Adult Members

(INTERVIEWER: Only record the details of household members of age 18 years and above.)

A1. Household member code/ ID ઘરના સભ્યનો કોડ/આઇડી	A2. Name નામ	A3. Relationship to the household head ઘરના વડા સાથેનો સંબંધ	A4. Age in years ઉંમર વર્ષમાં	A5. Gender Male -1, Female - 2, Other - 3 લિંગ પુરુષ - 1, સ્ત્રી -2, અન્ય- 3	A6. Highest Education Level Completed છેલ્લો પાસ કરેલ અભ્યાસ	A7. Marital Status વૈવાહિક દરજ્જો 1= Married (લગ્ન કર્યા) 2= Widowed/Widower (વિધવા) 3= Unmarried (અપરિણીત) 4= Divorced/Separated (છૂટાછેડા/અલગ)	A8. Current Employment Status હાલની રોજગાર સ્થિતિ 1=Parttime (3-4 hours) (પાર્ટટાઇમ (3-4 કલાક)) 2= Fulltime (7-8 hours and above) (પૂર્ણ સમય (7-8 કલાક અને તેથી વધુ)) 0= Unemployed (બેરોજગાર)
01(HoH)(ઘરના વડા)		01					
02							
03							
04							
05							
06							
07							
08							
09							
10							
11							
12							
13							
14							
15							

Block B: Household Roster for Children

(INTERVIEWER: Only record the details of household members of age below 18 years.)

B1. Child ID .બાળ કનો આઇ ડી	B2. Name of the child બાળકનું નામ	B3. Relationship to the household head ઘરના વડા સાથેનો સંબંધ	B4. Age in years (For under 1, write 0) ઉંમર વર્ષમાં (1 વર્ષથી નીચેની વયના બાળક માટે 0 લખો)	B5. Gender Male = 1 Female = 2 Other = 3 લિંગ પુરુષ = 1 સ્ત્રી = 2 અન્ય = 3	B6. Mother Line no. (From Block B) માતાનો લાઇન નંબર (B બ્લોકમાંથી)	B7. Father Line no. (From Block B) પિતાનો લાઇન નંબર (B બ્લોકમાં થી)	Ask only for 5 years or older. ફક્ત 5 વર્ષ કે તેથી વધુ વયના બાળકોને પૂછો			
							B8. Highest Education level complete d. છેલ્લો પાસ કરેલ અભ્યાસ	B9. Current employment status હાલની રોજગાર સ્થિતિ 1=Parttime (3-4 hours) (પાર્ટટાઇમ (3-4 કલાક)) 2= Fulltime (7-8 hours and above) (પૂર્ણ સમય (7-8 કલાક અને તેથી વધુ)) 0= Unemployed (બેરોજગાર)	B.10 Currently enrolled in school? Yes = 1 No = 2 હાલમાં શાળામાં નામ નોંધાવેલ છે? હા = 1, ના = 2	B.11 Type of school Govt. – 1, NGO – 2, Private – 3 શાળાનો પ્રકાર સરકારી – 1, એનજીઓ – 2, ખાનગી – 3
1										
2										
3										
4										
5										
6										
7										
8										
9										
10										

Code for A3 & B3			
1	Head	6	Grandparent
2	Wife/Husband	7	Father/Mother
3	Son/Daughter	8	Brothers/Sister
4	Daughter-in-law or Son-in law	9	Brother-in-law/ Sister-in-law
5	Grandchildren	99	Servant/Other

Code for A6 & B8			
1	No education	5	ITI Diploma
2	Primary Education (till class 5)	6	College Graduate
3	High School (class 6-10)	7	Post-Graduate
4	Higher Secondary (class 11-12)	9	Other (Specify) ____________

Block C: Drinking Water Availability, Usage and Storage (બ્લોક C: પીવાના પાણીની ઉપલબ્ધતા, વપરાશ અને સંગ્રહ)

Q No	Question	Response	Code
C1	What is your <u>main source</u> of drinking water?<br>તમારા પીવાના પાણીનો <u>મુખ્ય સ્ત્રોત</u> ક્યો છે?	Public Tap (જાહેર નળ)	1
		Neighbor's tap (પાડોશમાં નળ)	2
		Own house tap (government supplied)<br>પોતાના ઘરમાં નળ (સરકારે આપેલ)	3
		Well(કૂવો)	4
		Groundwater/Borewell/Tubewell/Handpump<br>(ભૂગર્ભજળ/બોરવેલ/ટ્યુબવએલ/હેન્ડપંપ)	5
		Government Truck/Government Tank<br>(સરકારી ટ્રક / સરકારી ટેન્ક)	6
		River/Canal/Lake/Pond/Reservoir(નદી/કેનાલ/સરોવર/તળાવ /પાણીનો ટાંકો)	7
		Other (specify)<br>(અન્ય (સ્પષ્ટ લખો))_________________	9
C2	What is your secondary source of drinking water?<br>તમારા પીવાના પાણીનો ગૌણ સ્ત્રોત શું છે?	Public Tap (જાહેર નળ)	1
		Neighbor's tap (પાડોશમાં નળ)	2
		Own house tap (government supplied)<br>પોતાના ઘરમાં નળ (સરકારે આપેલ)	3
		Well(કૂવો)	4
		Groundwater/Borewell/Tubewell/Handpump<br>(ભૂગર્ભજળ/બોરવેલ/ટ્યુબવએલ/હેન્ડપંપ)	5
		Government Truck/Government Tank<br>(સરકારી ટ્રક / સરકારી ટેન્ક)	6
		River/Canal/Lake/Pond/Reservoir(નદી/કેનાલ/સરોવર/તળાવ /પાણીનો ટાંકો)	7
		Other (specify)(અન્ય (સ્પષ્ટ લખો))	9
		None (કોઈ નહિ)	99
C3	How many times do you get water in a day?<br>દિવસમાં તમે કેટલી વાર પાણી ભરવા જાઓ છો?	All day long(આખો દિવસ)	1
		Once a day(દિવસમાં એક વાર)	2
		Twice a day(દિવસમાં બે વાર)	3
		Other(અન્ય)	4
C4	How many hours do you get water in a day?<br><br>Instruction: C4 will be asked if C3 is coded other than 1. Else skip to C5.	[ ]<br>No. of Hours<br>(Range- 1-12)	
C5	How far is the main source of drinking water from your dwelling?<br>પીવાના પાણીનો મુખ્ય સ્ત્રોત તમારા રહેઠાણથી કેટલે દૂર છે?	Within Premises (ઘરની અંદર)	1
		Less than 0.5 KM (અડધા કિમી કરતા ઓછું)	2
		0.5 to 1 KM (અડધાથી 1 કિમી)	3
		More than 1 KM (1 કિમી કરતા વધારે)	4
C6	Do you store water?	Yes, indoor (હા, ઇન્ડોર	1

Q No	Question	Response	Code
	(શું તમે પાણીનો સંગ્રહ કરો છો?)<br><br>Instruction: If coded 4 then skip to C9	Yes, outdoor (હા, આઉટડોર)	2
		Yes, both (હા, બંને)	3
		No(ના)	4

C7	Water Storage Tanks	C7.1 How do you store water?<br>Yes=1<br>No=2	C7.2 How often do you fill them in a day?<br>Once=1<br>Twice=2<br>Thrice=3<br>Four Times=4<br>None=5<br>Other=6	C7.3 How often do you get the water tank cleaned in one year?<br>Once=1<br>Twice=2<br>Thrice=3<br>Four times=4<br>More than four times=5<br>Not on yearly basis=6	C7.4 Instruction for Interviewer: Check and Verify before recording the response. How do you cover them?<br>Covered fully using a lid=1<br>Covered partially using a lid=2<br>Covered with plastic sheet=3<br>Covered with metal sheet=4<br>Covered with a cloth=5<br>Not covered=6	C7.5 Can mosquitoes get inside these sources of water storage that you use?<br>Yes=1<br>No=2
	પાણી સંગ્રહ ટાંકીઓ	તમે પાણીનો સંગ્રહ કેવી રીતે કરો છો?<br>હા=1<br>ના=2	તમે તેમને દિવસમાં કેટલી વાર ભરો છો?<br>એકવાર=1<br>બે વાર=2<br>ત્રણ વખત=3<br>ચાર વખત=4<br>કોઈ નહીં=5<br>અન્ય=6	તમે વર્ષમાં કેટલી વાર પાણીનો ટાંકો સાફ કરો છો?<br>એક વાર=1<br>બે વાર=2<br>ત્રણ વાર=3<br>ચાર વાર=4<br>ચાર કરતા વધારે વાર=5	(તપાસ અને ચકાસવા માટે ગણતરીકર્તા માટે નોંધ)<br>તમે તેમને કેવી રીતે આવરી લેશો?<br>ઢાંકણનો ઉપયોગ કરીને પૂરેપૂરો ઢાંકી રાખો છો=1<br>ઢાંકણનો ઉપયોગ કરીને અડધો-પડધો ઢાંકી રાખો છો=2<br>પ્લાસ્ટિકના કવરનો ઉપયોગ કરીને ઢાંકી રાખો છો=3<br>ધાતુના પતરાનો ઉપયોગ કરીને ઢાંકી રાખો છો=4<br>એક કાપડ સાથે આવરી લેવામાં=5<br>ઢાંકતા નથી=6	શું તમે ઉપયોગ કરો છો તે પાણીના સંગ્રહના આ સ્ત્રોતોમાં મચ્છર પ્રવેશી શકે છે?<br>હા=1<br>ના=2
1	Overhead water tank (ઓવરહેડ પાણીની ટાંકી)					
2	Underground water tank (ભૂગર્ભ પાણીની ટાંકી)					
3	Cement Tank (સિમેન્ટ ટાંકી)					
4	Buckets (ડોલમાં)					
5	Utensils (વાસણોમાં)					
6	Clay pot (માટીના ઘડામાં)					
7	Gallon(ગેલનમાં)					
8	Plastic can (પ્લાસ્ટિકના કેનમાં)					
9	Other (અન્ય)					

Q No	Question	Response	Code
C8	How many water containers do you use to store water in a day?<br>તમે દિવસમાં કેટલી ડોલ પાણીનો સંગ્રહ કરો છો?	[ ]<br>(Number of Containers)<br>(Range- 1- 10)	
C9	Do you filter/boil/purify water for drinking purposes and do you store water after purification?<br>તમે પીવાના પાણી માટે ગાળવાની/શુધ્ધિકરણની પ્રક્રિયા કર્યા પછી પાણીનો સંગ્રહ કરો છો?<br>શું તમે પીવાના હેતુ માટે પાણીને ફિલ્ટર/ઉકાળો/શુધ્ધ કરો છો?	Filter or purify drinking water and store	1
		Filter or purify drinking water but do not store	2
		Do not filter or purify drinking water but store	3
		Do not filter or purify drinking water and do not store	4
C10	How do you store the filtered water?<br>તમે ગાળેલા પાણીનો સંગ્રહ કેવી રીતે કરો છો?<br><br>[Record All that Apply]<br>[જે લાગુ પડે છે તે બધું પસંદ કરો]	Water bottles with tight caps/lids (ચૂસ્ત રીતે બંધ થતી/ઢાંકણવાળી બોટલમાં પાણી ભરીને)	1
		Water bottles without caps/lids (ઢાંકણ/આવરણ વિનાની બોટલમાં પાણી ભરીને)	2
		Utensils with metal screens/plates coverings (વાસણોને ધાતુના પતરા/પ્લેટ વડે ઢાંકીને)	3
		Utensils without any coverings (કોઈપણ જાતના ઢાંકણ વિનાના વાસણો)	4
		Other sources with covering (ઢાંકણ સાથેના અન્ય સાધનો)	5
		Other sources without coverings (ઢાંકણ વિનાના અન્ય સાધનો)	6
C11	How often do you clean these sources of water storage such as buckets, bottles, gallons, cans etc. with soap?<br>ડોલ, બોટલ, ગેલન, કેન વગેરે જેવા પાણી સંગ્રહના સાધનો તમે કેટલી વાર સાબુથી સાફ કરો છો?	Daily(દરરોજ)	1
		More than once a week (અઠવાડિયે એક કરતા વધુ વાર)	2
		Once a week (અઠવાડિયે એક વાર)	3
		Once a month (મહિને એક વાર)	4
		Once in two months (બે મહિને એક વાર)	5
		Once in three months (ત્રણ મહિને એક વાર)	6
		Once in six months (છ મહિને એક વાર)	7
		Once a year (વર્ષે એક વાર)	8
		Never cleaned (ક્યારેય સાફ કરતા નથી)	9
C12	In the last month how much money did your household spend for water (including municipality water bill, money spent on drinking water supply etc.)?<br>છેલ્લા 30 દિવસમાં તમારા પરિવારે પાણી માટે કેટલા રૂપિયાનો ખર્ચ કર્યો હતો (મ્યુનિસિપાલિટીના પાણી બિલ, પીવાના પાણીના સપ્લાય વગેરે માટે કરેલ ખર્ચ સહિત)?	[ ]<br>Money Spent on Drinking Water	

Block D: Time-use information (બ્લોક D: સમયના વપરાશની માહિતી)

Time used in activities પ્રવૃત્તિઓમાં વપરાયેલ સમય		In Minutes મિનિટ્સ માં
D1	Total time used in fetching water by family members in the last 3 days.<br>છેલ્લા 3 દિવસમાં તમારા પરિવારના સભ્યો દ્વારા પાણી ભરવા પાછળ વપરાયેલ કુલ સમય	
D2	Number of times children (< 18) fetched water in the past 3 days.<br>છેલ્લા 3 દિવસમાં બાળકો (18થી ઓછી વયના) કેટલી વખત પાણી ભરવા માટે ગયા હતા?	
D3	Number of times adult men fetched water in the past 3 days.<br>છેલ્લા 3 દિવસમાં પુખ્ય વયના પુરુષો કેટલી વખત પાણી ભરવા માટે ગયા હતા?	
D4	Number of times adult women fetched water in the past 3 days.<br>છેલ્લા 3 દિવસમાં પુખ્ય વયની સ્ત્રીઓ કેટલી વખત પાણી ભરવા માટે ગઈ હતી?	
D5	Total time spent on purifying (e.g., filtering or boiling) water by family members in the last 3 days.<br>છેલ્લા 3 દિવસમાં તમારા પરિવારના સભ્યો દ્વારા પાણી શુધ્ધ કરવા માટે (દા.ત. પાણી ગાળવું કે શુધ્ધ કરવું) વપરાયેલ કુલ સમય	

Block E: Basic Health and Hygiene Questionnaire (બ્લોક E: સામાન્ય આરોગ્ય અને શરીર સ્વચ્છતા અંગેની પ્રશ્નોત્તરી)

Health Questions (short-term morbidity) (આરોગ્ય પ્રશ્નો (ટૂંકા-ગાળાની બિમારી))		E1: Did anyone in the family get sick in the last 90 days due to this illness? છેલ્લા 90 દિવસમાં તમારા પરિવારની કોઇ વ્યક્તિ આ રોગને લીધે બિમાર પડી છે?		E2: Household member code/ ID (A1 & B1 from block A & B) ઘરના સભ્યનો કોડ / આઇડી (બ્લોક B માંથી B1)	E3: Did they seek treatment? શું તેઓએ બિમારીની સારવાર લીધી હતી? Yes-1, No-2 **હા**-1, **ના**-2	E4: Were they hospitalized? શું તેમને હોસ્પિટલમાં દાખલ કરાયા હતા? Yes-1, No-2 **હા**-1, **ના**-2	E5: How much expenditure was incurred in total for treatment? સારવાર માટે કુલ કેટલો ખર્ચ થયો હતો?
		Yes હા	No ના				
1	Malaria મેલેરિયા	1	2				
2	Diarrhea ડાયરીયા (ઝાડા)	1	2				
3	Vomiting વોમિટીંગ (ઉલ્ટી)	1	2				
4	Fever or Cough તાવ કે શરદી-કફ	1	2				
5	Dengue ડેંન્ગ્યૂ	1	2				
6	Chikungunya ચિકનગુનિયા	1	2				
7	Stomach ache or other stomach problems પેટનો દુ:ખાવો અથવા પેટની અન્ય કોઇ બિમારી	1	2				

E6	In the last seven days, did you and your family used soap while washing hand? શું તમે અને તમારો પરિવાર હાથ ધોવા માટે સાબુનો ઉપયોગ કરો છો?	Every time (દર વખતે)	1
		Sometimes (ક્યારેક)	2
		Rarely (ભાગ્યે જ)	3
		Never (ક્યારેય)	4
E7	In the last seven days, did you and your family members bathe every day? શું તમે અને તમારા પરિવારના સભ્યો દરરોજ સ્નાન કરો છો?	Everyone bathed everyday	1
		Some members bathed everyday	2
		Everyone bathed but only on somedays	3
		Other	4

E8	In last month how much money did your family spend on personal hygiene products (e.g., soap and shampoo)?<br>છેલ્લા 30 દિવસ દરમિયાન તમારા પરિવારે વ્યક્તિગત સ્વચ્છતાને લગતી ચીજવસ્તુઓ (દા.ત. સાબુ અને શેમ્પૂ) પાછળ કેટલો ખર્ચ કર્યો હતો?	[ ]<br>Enter 0 if No Money was Spent.<br>જો કોઈ ખર્ચ કર્યો ન હોય તો 0 લખો
E9	In last month how much money did your family spend on household cleaning products (e.g., Phenyl)?<br>છેલ્લા 30 દિવસ દરમિયાન તમારા પરિવારે સફાઈને લગતી ચીજવસ્તુઓ (દા.ત. ફિનાઇલ) પાછળ કેટલો ખર્ચ કર્યો હતો?	[ ]<br>Enter 0 if No Money was Spent.<br>જો કોઈ ખર્ચ કર્યો ન હોય તો 0 લખો

Q No	Question	Response	Code
E10	In your opinion, how big is the problem of mosquitoes in your community or neighborhood?<br>તમારા મતે, તમારા વિસ્તારમાં કે આજુબાજુમાં મચ્છરોનો ઉપદ્રવ કેટલા મોટા પ્રમાણમાં છે?	No mosquitoes (મચ્છરો નથી)	1
		Very few mosquitoes (બહુ ઓછા મચ્છરો છે)	2
		Some mosquitoes (થોડા પ્રમાણમાં મચ્છરો છે)	3
		A lot of mosquitoes (ઘણા પ્રમાણમાં મચ્છરો છે)	4
E11	Are any of these measures taken to tackle the problem of mosquitoes in your community or neighborhood?<br>તમારા વિસ્તારમાં કે આજુબાજુમાં મચ્છરોના ઉપદ્રવના નિવારણ માટે આમાંના કોઈ પગલાં લેવામાં આવે છે?<br><br>[Record All that Apply]<br>[જે લાગુ પડે છે તે બધું પસંદ કરો]	Public spraying by Government (સરકાર દ્વારા સાર્વજનિક રીતે દવાનો છંટકાવ)	1
		Private spraying by your housing association/group (તમારી હાઉસીંગ સોસાયટી/સમૂહ દ્વારા ખાનગી ધોરણે દવાનો છંટકાવ)	2
		Personal household spraying (વ્યક્તિગત રીતે ઘરમાં દવાનો છંટકાવ)	3
		Larvicides (મચ્છરોના લારવા પર દવા છાંટી તેનો નાશ કરવો)	4
		None	5
		Other Sources (Please Specify) (અન્ય કોઈ સાધન હોય તો એ જણાવો)	9
E12	In your opinion, how big is the problem of mosquitoes inside your house or dwelling unit?<br>તમારા મતે, તમારા ઘરમાં અથવા રહેઠાણની જગ્યાની અંદર મચ્છરોનો ઉપદ્રવ કેટલા મોટા પ્રમાણમાં છે?	No mosquitoes (મચ્છરો નથી)	1
		Very few mosquitoes (બહુ ઓછા મચ્છરો છે)	2
		Some mosquitoes (થોડા પ્રમાણમાં મચ્છરો છે)	3
		A lot of mosquitoes (ઘણા પ્રમાણમાં મચ્છરો છે)	4
E13	Do you use the following to prevent mosquito bites in your house or dwelling unit?<br>તમારા ઘરમાં કે રહેઠાણની જગ્યામાં મચ્છરોના કરડવાથી બચવા તમે નીચેનામાંથી કોનો ઉપયોગ કરો છો?<br><br>[Record All that Apply]<br>[જે લાગુ પડે છે તે બધું પસંદ કરો]	Cover doors and windows with wire mesh/screens (બારી અને દરવાજાને વાયરની બારીક જાળી/પડદા વડે ઢાંકીએ છીએ)	1
		Use mosquito nets (મચ્છરદાનીનો ઉપયોગ કરીએ છીએ)	2
		Apply insect repellant (મચ્છર ભગાવવાની અગરબત્તીનો ઉપયોગ કરીએ છીએ)	3
		Mosquito coils or oils (e.g., All Out) (મસ્કિટો કોઇલ અથવા ઓઇલ્સ (ઓલ આઉટ વગેરે)	4

Q No	Question	Response	Code
		Others(અન્ય)	9
E14	What is the condition of the screen used to cover the windows? (Enumerator can notice and report)<br>બારીઓને ઢાંકવા ઉપયોગમાં લેવાતા પડદાની સ્થિતિ કેવી છે? (સંગણક અવલોકન કરીને નોંધ કરી શકે છે)<br><br>Instruction for Interviewer: Observe before recording the response.	Good condition screens and fully covers all windows (પડદાની સ્થિતિ સારી છે અને બધી બારીઓને સંપૂર્ણપણે ઢાંકે છે)	1
		Good condition screens but doesn't fully cover all windows (પડદાની સ્થિતિ સારી છે પણ બધી બારીઓને સંપૂર્ણપણે ઢાંકતા નથી)	2
		Broken/tearing (તૂટેલા/ફાટી ગયેલા છે)	3
		Not applicable (no screen) (લાગું પડતું નથી (પડદા નથી))	9
E15	In the last month on how many days did you use these preventive measures (especially use mosquito nets) for all family members?<br>છેલ્લા મહિનાના કેટલા દિવસોમાં તમે તમારા પરિવારના બધા સભ્યો માટે આ નિવારક પગલાંઓનો ઉપયોગ કર્યો હતો (ખાસ કરીને મચ્છરદાનીનો ઉપયોગ)	None (એક પણ નહીં)	1
		Less than 5 days (5 કરતા ઓછા દિવસ)	2
		5 to 10 days (5 થી 10 દિવસ)	3
		11-20 days (11 - 20 દિવસ)	4
		21-29 days (21 - 29 દિવસ)	5
		All 30 days (તમામ 30 દિવસ)	6
E16	Is your house close to cattle shed or a barn?<br>શું તમારું ઘર ઢોર રાખવા માટેના શેડ કે ગમાણની નજીક છે?	Yet. It is a part of my residence (હા.... તે મારા રહેઠાણનો એક ભાગ છે)	1
		Yes. it is in the neighborhood (હા. તે નજીકમાં જ આવેલ છે)	2
		No(ના)	3

Block F: Housing Condition (બ્લોક F: ઘરની સ્થિતિ)

Q No	Question	Response	Code
F1	Is this house owned or rented?<br>આ ઘર તમારું પોતાનું છે કે ભાડાનું?	Owned (પોતાનું)	1
		Rented/ Leased (ભાડાનું/ભાડા પટ્ટાનું)	2
		Other (specify) (અન્ય (સ્પષ્ટ લખો)	9
F2	How many rooms are there in this house?<br>Interviewer Instruction: Include Kitchen.<br>ઘરમાં કેટલા રૂમ છે? (ગણક માટે નોંધ: રસોડું શામેલ કરો)	No of Rooms<br>(Range- 1-10)	
F3	Is there a separate room used as kitchen?<br>રસોડા તરીકે ઉપયોગ લેવા માટે અલગ રૂમ છે?	Yes(હા)	1
		No(ના)	2
F4	What is the type of housing construction?<br>ઘરનું બાંધકામ કેવા પ્રકારનું છે?<br>Interviewer Instruction: Please observe and record. Do not ask.<br>(કૃપા કરી અવલોકન કરીને નોંધો. પૂછશો નહીં)	Pucca(પાકું)	1
		Semi-pucca(અર્ધ-પાકું)	2
		Kuccha(કાચું)	3
F5	What is the predominant roof type?<br>છાપરું મોટા ભાગે શાનું બનેલું છે?<br><br>Interviewer Instruction: Please observe and record. Do not ask.	Grass, Thatch, Mud, Wood (ઘાસ, પરાળ, માટી, લાકડું વગેરેનું)	1
		Tile(નળિયાનું)	2
		Slate (છાપરા પર ઢાંકવાના પતરા કે ટાઇલ્સનું)	3
		Plastic(પ્લાસ્ટિકનું)	4
		GI Metal, Asbestos (જીઆઇ મેટલ, એસ્બેસ્ટોસના પતરાનું)	5
		Cement/ Concrete (સિમેન્ટ/ કોન્ક્રીટ)	6
		Brick(ઈંટો)	7
		Stone(પથ્થર)	8
		Other(અન્ય)	9
F6	How many floors does your house have?<br>તમારું ઘર એક માળનું છે કે એકથી વધુ માળનું?	One Ground Floor, Single Storey (એક માળનું)	1
		Two Floors (in same unit with one entrance) (બે માળનું (ઘરમાં એક જ દરવાજો હોય એવું))	2
		Two separate Floors (બે અલગ-અલગ માળનું)	3
		Three Floors (ત્રણ માળનું)	4
		More than Three Floors (ત્રણ કરતા વધારે માળનું)	5
F7	What is the primary source of lighting in your dwelling?<br>તમારા ઘરમાં લાઇટની વ્યવસ્થાનો મુખ્ય સ્રોત ક્યો છે?	Electricity(વીજળી)	1
		Kerosene(કેરોસીન)	2
		Other Oil (અન્ય બળતણ તેલ)	3
		Gas(ગેસ)	4
		Candle(દીવાબત્તી/ફાનસ)	5

Q No	Question	Response	Code
		Other (specify) (અન્ય (સ્પષ્ટ લખો))	9
F8	What is the primary source of cooking fuel in your dwelling?<br>તમારા ઘરમાં રસોઇ બનાવવા માટેના બળતણનો મુખ્ય સ્રોત ક્યો છે?	LPG/ Natural Gas (એલપીજી/કુદરતી ગેસ).	1
		Electricity(વીજળી)	2
		Biogas(બાયોગેસ)	3
		Kerosene(કેરોસીન)	4
		Coal/Lignite/Charcoal (કોલસા/ખનીજ કોલસા/લાકડીયા કોલસા)	5
		Wood (primus)/ Straw/Shrubs/Grass (લાકડા (પ્રાઇમસ) / સાઠી /છોડના ઈંધણા / ઘાસપાંદડા)	6
		Agricultural Crop (Residue) (ખેતપાકોનો વધેલો ભાગ (પરાળ)).	7
		Animal Dung (પશુઓના છાણમાંથી બનતા છાણા)	8
		Other (specify) (અન્ય (સ્પષ્ટ લખો))	9
F9	What is the main type of toilet that you use?<br>ઘરમાં મુખ્યત્વે ક્યા પ્રકારના શૌચાલયનો ઉપયોગ કરવામાં આવે છે?<br>If coded 1, skip to next section Block G<br>જો ઉપરના જવાબનો કોડ 1 છે તો બ્લોક G છોડી દો	No Toilet/ Field (શૌચાલય નથી/ખુલ્લામાં)	1
		Septic Tank/ Pit Toilet (ખાળ કૂવા / ખાડા જાજરૂ)	2
		Flush Latrine (પાણીની સગવડવાળું જાજરૂ)	3
		Other (specify) (અન્ય (સ્પષ્ટ લખો))	9
F10	With how many other households does your household share a toilet?<br>તમારા ઘરના લોકો જે શૌચાલયનો ઉપયોગ કરે છે એ બીજા કેટલા ઘરો દ્વારા ઉપયોગમાં લેવામાં આવે છે?<br>Instruction: Ask this question if F9 is coded other than 1.	[ ]<br>Enter 0 if toilet is not shared<br>(જો અલાયદું શૌચાલય હોય તો 00 લખો)	

Block G: Household Characteristics (બ્લોક G: ઘર-પરિવારની લાક્ષણિકતાઓ)

Q No	Question	Response	Code
G1	To which category does the household head belong?<br>(ઘરના વડા કઈ કેટેગરીમાં આવે છે?)	Scheduled Tribe (અનુસૂચિત જનજાતિ)	1
		Scheduled Caste (અનુસૂચિત જાતિ)	2
		Other Backward Class (અન્ય પછાત વર્ગ)	3
		General (સામાન્ય)	4
		Don't Know (ખબર નથી)	5
		Don't want to reveal (જાહેર કરવા માંગતા નથી)	6
G2	What is the household head's Religion?<br>(ઘરના વડાનો ધર્મ ક્યો છે?)	Hinduism (હિન્દુ)	1
		Islam (ઇસ્લામ)	2
		Christianity (ખ્રિસ્તી)	3
		Buddhism (બૌધ્ધ)	4
		Sikhism (શિખ)	5
		Don't know (ખબર નથી)	6
		Don't want to reveal (જાહેર કરવા માંગતા નથી)	7
		Other (specify) (અન્ય (સ્પષ્ટ લખો))	9
G3	What is the <u>main source of income</u> of the household?<br>ઘરની આવકનો મુખ્ય સ્રોત ક્યો છે?	Own Cultivation (પોતાની ખેતી)	1
		Agricultural wage labor(દૈનિક ખેત મજૂરી)	2
		Non-agricultural wage labor(બિન-ખેત મજૂર)	3
		Trade/ business(વ્યાપાર / ધંધો)	4
		Salaried employment(પગારદાર કર્મચારી)	5
		Pension/ rent/ dividend (પેન્સન/ભાડા/ડિવિડન્ડની આવક)	6
		Self-employment (સ્વ-રોજગાર)	7
		Other (specify)<br>અન્ય (સ્પષ્ટ લખો)	9
G4	How many years has your family lived in this city?<br><br>(તમારો પરિવાર આ શહેરમાં કેટલા વર્ષથી રહે છે?)	[ ]<br><br>Enter 90 if more than 90 Years (or "forever")<br>(જો 90 વર્ષ કરતા વધારે (અથવા "કાયમી") છે તો 90 લખો)	
G5	Where did your family come from?<br>(તમારો પરિવાર ક્યાંથી આવ્યો હતો?)<br><br>Instruction: A4 will be asked if A3 is coded other than 90	Same city (આ જ શહેરમાંથી)	1
		Not same city but same state (આ શહેરમાંથી નહિ પણ આ રાજ્યમાંથી)	2
		Not same state but same country (આ રાજ્યમાંથી નહિ પણ આ દેશમાંથી)	3
		Another country (બીજા કોઈ દેશમાંથી)	9
G6		Contact 1	Contact 2

<table>
<tr><td rowspan="2"></td><td rowspan="2">What are the names and best contact phone numbers of two of your relatives/ neighbors/ friends who could help us reach you in case you move in the next few years?
જો તમે આગામી થોડા વર્ષોમાં બીજે ક્યાંય જતા રહેવાના હો તો તમારો જલ્દીથી સંપર્ક કરી શકાય એવા કેટલાક તમારા સંબંધીઓ / પાડોશીઓ / મિત્રો પૈકી કોઈ બે વ્યક્તિના નામ અને ફોન નંબર શું છે?</td><td>Name
(નામ):</td><td></td><td></td></tr>
<tr><td>Phone No.
(ફોન નંબર):</td><td></td><td></td></tr>
</table>

Block H: Household Assets (બ્લોક H: ઘરની મિલકતો)

	INTERVIEWER: Regarding household durables, record Yes or No<br>(ઈન્ટરવ્યુઅર: ઘરવપરાશની ટકાઉ ચીજવસ્તુઓ અંગે હા અથવા ના પર કુંડાળું કરો)	**A.** Does the household have any of the following items?<br>**A.** શું ઘરમાં નીચેનામાંથી કોઇ ચીજવસ્તુઓ છે?		B. Please list the number of items owned here.<br>(Answer only if coded 1 in column A)<br>કૃપા કરી અહીં ઘરમાં વસાવેલ ચીજવસ્તુઓની યાદીબનાવો<br>(જો કોલમ A માં કોડ 1 આપેલ હોય તો જ જવાબ આવશે)
		Yes<br>હા	No<br>ના	
H1	Telephone / mobile phone ટેલીફોન / મોબાઇલ ફોન	1	2	
H2	Clock or watch દિવાલ ઘડિયાળ અથવા કાંડા ઘડિયાળ	1	2	
H3	Electric fan ઈલેક્ટ્રિક પંખો	1	2	
H4	Black and white television set બ્લેક એન્ડ વ્હાઇટ ટેલીવિઝન સેટ	1	2	
H5	Color television set કલર ટેલીવિઝન સેટ	1	2	
H6	VCR/VCP/DVD/VCD player વીસીઆર/વીસીપી/ડીવીડી/વીસીડી પ્લેયર	1	2	
H7	Radio, transistor, music speakers or stereo રેડિયો, ટ્રાન્ઝિસ્ટર, સંગીત બોલનારા કે સ્ટીરિયો	1	2	
H8	Sewing machine સિલાઈ મશીન)	1	2	
H9	Air conditioner (એર કન્ડિશનર)	1	2	
H10	Refrigerator/ Fridge (રેફ્રીજરેટર/ ફ્રીજ)	1	2	
H11	Washing machine (વૉશીંગ મશીન)	1	2	
H12	Mixer/ grinder (મિક્ષ્ચર / ગ્રાઈન્ડર)	1	2	
H13	Car or jeep (કાર અથવા જીપ)	1	2	
H14	Bicycle (બાઇસિકલ)	1	2	
H15	Auto rickshaw, Motorcycle, Moped or Scooter (ઓટો રીક્ષા, મોટરસાયકલ, મોપેડ કે સ્કૂટર)	1	2	
H16	Bullock cart (બળદગાડું)	1	2	
H17	Thresher (થ્રેસર)	1	2	
H18	Tractor (ટ્રેક્ટર)	1	2	
H19	Well /Tubewell (કૂવો / ટ્યુબવેલ)	1	2	
H20	Grain storage can (અનાજ સંગ્રહ ટાંકી)	1	2	
H21	Pressure Cooker (પ્રેસર કૂકર)	1	2	
H22	Chair/stool (ખુરશી / સ્ટૂલ)	1	2	
H23	Cot or Bed (પલંગ કે ખાટલો)	1	2	
H24	Table (ટેબલ)	1	2	
H25	Jewelry (ઘરેણા)	1	2	
H26	Bank account/ post office account (બેંકમાં ખાતું / પોસ્ટ ઑફિસમાં ખાતું)	1	2	
H27	Other Assets (અન્ય મિલકતો<br>(specify) ((સ્પષ્ટ લખો)) ______________________	1	2	
H28	Does your household own any land?		Yes(હા)	1

	શું તમારા ઘરની માલિકીની કોઈ જમીન છે?<br><br>Instruction: If coded 2, Skip to END.	No(ના)	2
H29	How much land does your household own?<br>કેટલી જમીન તમારા ઘરની માલિકીની છે?<br><br>Instruction: Ask if H28 coded 1	(Land in ACRES(એકર))<br>Land unit (record actual response as is here) ___________________<br>જમીન એકમ (સાચો પ્રતિભાવ અહીં નોંધો)	
H30	How much cultivable land does your household own? ખેતીની કેટલી જમીન તમારા ઘરની માલિકીની છે?<br>Instruction: Ask if H28 coded 1	(Land in ACRES(એકર))<br>Land unit (record actual response as is here) ___________________<br>જમીન એકમ (સાચો પ્રતિભાવ અહીં નોંધો)	
