# Supplementary material for: Socioeconomic and household water management determinants of malaria and other vector-borne disease prevention in Urban Gujarat, India
Source: Malar J. 2026 Feb 17;25:141. doi: 10.1186/s12936-026-05830-2 (PMC13015023; doi:10.1186/s12936-026-05830-2)
Supplement: Supplementary file 2 — Additional file 2. [file 12936_2026_5830_MOESM2_ESM.pdf]

Supplementary Appendix B: Bivariate regression results

Table B.1. Bivariate logistic regressions of predictors of outcome variables (Odds Ratio)

	(1) At least 1 disease case reported	(2) More than 1 symptom reported	(3) Active prevention measure used	(4) More than 3 prevention measures used
Demographics				
**Age of household head** (in years)	1.014	0.988**	0.993	0.992**
	(0.009)	(0.006)	(0.005)	(0.004)
**Education of household head**				
Below High School	Ref	Ref	Ref	Ref
High School and above	1.647*	2.276***	1.258	1.018
	(0.486)	(0.713)	(0.213)	(0.193)
**Family size**	1.235***	1.163***	0.932	0.942
	(0.085)	(0.068)	(0.045)	(0.057)
**Proportion of female members**	0.996	1.006	0.997	0.992***
	(0.007)	(0.004)	(0.003)	(0.002)
**Proportion of children**	0.997	1.020**	0.996	0.995
	(0.006)	(0.009)	(0.003)	(0.004)
**Wealth index**				
Bottom 20 percent (0-20%)	Ref	Ref	Ref	Ref
Bottom 40 percent (20-40%)	1.595	0.589	0.907	3.170***
	(0.576)	(0.214)	(0.211)	(0.761)
Bottom 60 percent (40-60%)	1.683	0.221**	1.479	4.291***
	(0.551)	(0.132)	(0.438)	(1.224)
Top 40 percent (60-80%)	0.944	0.747	1.533	3.943***
	(0.397)	(0.458)	(0.463)	(1.197)
Top 20 percent (80-100%)	4.880***	1.068	1.556	1.993*
	(1.599)	(0.605)	(0.437)	(0.725)
**Migrant**				
From the same city	Ref	Ref	Ref	Ref
Not from the same city	2.287*	0.344**	1.258	1.070
	(1.083)	(0.168)	(0.368)	(0.347)
**Caste**				
General	Ref	Ref	Ref	Ref
Scheduled Castes	0.890	7.366***	0.720	1.239
	(0.409)	(4.023)	(0.249)	(0.427)
Scheduled Tribes	1.088	3.010**	0.891	0.461**
	(0.501)	(1.502)	(0.260)	(0.141)
Other Backward Classes	0.580	1.605	0.772	0.507**
	(0.273)	(0.641)	(0.183)	(0.153)
**Religion**				
Majority (Hindu)	Ref	Ref	Ref	Ref
Others	0.597	0.352	1.730**	1.015
	(0.370)	(0.247)	(0.458)	(0.393)

Protection behaviors				
**Expenditure on cleanliness**	1.373*	0.929	0.852	1.171
	(0.248)	(0.246)	(0.136)	(0.149)
Water availability				
**Less water availability**				
Water available more than once a day	Ref	Ref	Ref	Ref
Water available only once a day	0.671	0.335**	1.880***	0.723
	(0.230)	(0.148)	(0.445)	(0.250)
**Relative frequency of fetching water**				
Low/below-average frequency	Ref	Ref	Ref	Ref
High/above-average frequency	0.974	2.590***	0.814	0.760
	(0.341)	(0.947)	(0.250)	(0.276)
Storage practices				
**Household purifies and stores water**	1.578	2.773***	0.470***	0.818
	(0.638)	(0.878)	(0.101)	(0.244)
**Water storage container type**				
None/Do not store	1.736	1.051	4.973***	0.462
	(1.265)	(1.086)	(2.560)	(0.310)
Only small	Ref	Ref	Ref	Ref
Both small and large or only large	1.736*	3.417***	1.256	1.807**
	(0.549)	(1.178)	(0.281)	(0.436)
**Large container is clean**				
Otherwise	Ref	Ref	Ref	Ref
At least 1 large container is clean	1.180	2.745***	1.264	2.053***
	(0.287)	(0.723)	(0.247)	(0.497)
**Small container is clean**				
Otherwise	Ref	Ref	Ref	Ref
At least 1 small container is clean	2.494***	1.113	0.886	0.782
	(0.635)	(0.483)	(0.182)	(0.214)
**Large container is impermeable**				
Large container is permeable	Ref	Ref	Ref	Ref
At least 1 large container is impermeable	0.749	2.794	0.563*	0.384***
	(0.321)	(2.022)	(0.175)	(0.119)
**Small container is impermeable**				
Small container is permeable	Ref	Ref	Ref	Ref
At least 1 small container is impermeable	1.175	1.559	0.901	0.934
	(0.629)	(0.671)	(0.239)	(0.246)

Standard errors clustered at the ward level. Robust standard errors in parentheses. Ref: reference category. *** p<0.01, ** p<0.05, * p<0.1

Table B.2. Bivariate logistic regressions of predictors of outcome variables (Odds Ratio)

	(5) More than 1 indoor measure used	(6) More than 1 outdoor measure used	(7) High risk perception of mosquitoes
Demographics			
**Age of household head** (in years)	0.998	0.987***	0.998
	(0.004)	(0.004)	(0.005)
**Education of household head**			
Below High School	Ref	Ref	Ref
High School and above	0.943	1.961***	0.669**
	(0.171)	(0.360)	(0.120)
**Family size**	0.986	0.783***	1.029
	(0.058)	(0.048)	(0.064)
**Proportion of female members**	0.996*	0.993***	0.996
	(0.002)	(0.002)	(0.003)
**Proportion of children**	1.002	0.989***	1.012***
	(0.003)	(0.004)	(0.003)
**Wealth index**			
Quintile 1: Bottom 20 percent (0-20%)	Ref	Ref	Ref
Quintile 2: Bottom 40 percent (20-40%)	1.337	3.459***	0.868
	(0.591)	(1.011)	(0.206)
Quintile 3: Bottom 60 percent (40-60%)	1.565	4.523***	0.908
	(0.592)	(1.492)	(0.265)
Quintile 4: Top 40 percent (60-80%)	0.732	5.798***	0.691
	(0.381)	(1.898)	(0.208)
Quintile 5: Top 20 percent (80-100%)	4.294***	4.740***	0.452**
	(1.766)	(1.817)	(0.143)
**Migrant**			
From the same city	Ref	Ref	Ref
Not from the same city	1.238	1.882	2.106**
	(0.354)	(0.753)	(0.704)
**Caste**			
General	Ref	Ref	Ref
Scheduled Castes	0.710	0.757	1.341
	(0.378)	(0.324)	(0.436)
Scheduled Tribes	1.243	0.265***	0.426**
	(0.676)	(0.107)	(0.164)
Other Backward Classes	0.491	0.309***	1.836***
	(0.317)	(0.116)	(0.382)
**Religion**			

Majority (Hindu)	Ref	Ref	Ref
Others	0.597	0.973	1.991*
	(0.370)	(0.336)	(0.706)
Protection behaviors			
**Expenditure on cleanliness**	0.989	1.438**	0.795*
	(0.130)	(0.211)	(0.101)
Water availability			
**Less water availability**			
Water available more than once a day	Ref	Ref	Ref
Water available only once a day	0.589	0.654	1.032
	(0.194)	(0.180)	(0.334)
**Relative frequency of fetching water**			
Low/below-average frequency	Ref	Ref	Ref
High/above-average frequency	0.946	1.619	1.128
	(0.337)	(0.514)	(0.392)
Storage practices			
**Household purifies and stores water**	1.023	0.810	0.366***
	(0.301)	(0.241)	(0.123)
**Water storage container type**			
None/Do not store	1.596	0.544	0.079***
	(0.631)	(0.327)	(0.050)
Only small	Ref	Ref	Ref
Both small and large or only large	2.510	4.997***	0.723
	(1.854)	(1.585)	(0.181)
**Large container is clean**			
Otherwise	Ref	Ref	Ref
At least 1 large container is clean	1.362	3.584***	0.981
	(0.303)	(0.850)	(0.193)
**Small container is clean**			
Otherwise	Ref	Ref	Ref
At least 1 small container is clean	0.868	1.474	1.595**
	(0.225)	(0.403)	(0.360)
**Large container is impermeable**			
Large container is permeable	Ref	Ref	Ref
At least 1 large container is impermeable	0.346***	0.352***	0.544
	(0.101)	(0.122)	(0.266)
**Small container is impermeable**			
Small container is permeable	Ref	Ref	Ref

At least 1 small container is impermeable	0.888	0.588**	0.593
	(0.199)	(0.154)	(0.219)

Standard errors clustered at the ward level. Robust standard errors in parentheses. Ref: reference category. *** p<0.01, ** p<0.05, * p<0.1
